# Akt1-dependent expression of angiopoietin 1 and 2 in vascular smooth muscle cells leads to vascular stabilization

**DOI:** 10.1038/s12276-022-00819-8

**Published:** 2022-08-05

**Authors:** Jung Min Ha, Seo Yeon Jin, Hye Sun Lee, Hye Jin Kum, Farzaneh Vafaeinik, Hong Koo Ha, Sang Heon Song, Chi Dae Kim, Sun Sik Bae

**Affiliations:** 1 Gene and Cell Therapy Research Center for Vessel-Associated Disease, Medical Research Institute, and Department of Pharmacology, Pusan National University School of Medicine, Yangsan, 50612 Republic of Korea; 2 Department of Urology, Pusan National University School of Medicine, Yangsan, 50612 Republic of Korea; 3 Department of Internal Medicine, Pusan National University School of Medicine, Yangsan, 50612 Republic of Korea

**Keywords:** Growth factor signalling, Atherosclerosis

## Abstract

Retinal angiogenesis was delayed in VSMC-specific Akt1-deficient mice (Akt1^∆SMC^) but not in Akt2^∆SMC^ mice. The proliferation of ECs, recruitment of pericytes, and coverage of VSMCs to the endothelium were defective in Akt1^∆SMC^. The silencing of Akt1 in VSMCs led to the downregulation of angiopoietin 1 (Ang1) and the upregulation of Ang2. The activation of Notch3 in VSMCs was significantly reduced in the retinas of Akt1^∆SMC^ mice. Silencing Akt1 suppressed the activation of Notch3. Moreover, the silencing of Notch3 downregulated Ang1, whereas the overexpression of Notch3 intracellular domain (NICD3) enhanced Ang1 expression. The nuclear localization and transcriptional activity of yes-associated protein (YAP) were affected by the expression level of Akt1. Silencing YAP downregulated Ang2 expression, whereas overexpression of YAP showed the opposite results. Ang1 antibody and Ang2 suppressed endothelial sprouting of wild-type aortic tissues, whereas the Ang2 antibody and Ang1 facilitated the endothelial sprouting of aortic tissues from Akt1^∆SMC^ mice. Finally, severe hemorrhage was observed in Akt1^∆SMC^ mice, which was further facilitated under streptozotocin (STZ)-induced diabetic conditions. Therefore, the Akt1-Notch3/YAP-Ang1/2 signaling cascade in VSMCs might play an essential role in the paracrine regulation of endothelial function.

## Blood vessel formation: Nearly identical proteins but different effects

Proteins Akt1 and Akt2 are nearly identical, but they play different roles in angiogenesis, the formation of new blood vessels. Angiogenesis is crucial in wound healing and development, and also in tumor formation. Although Akt1’s role in angiogenesis is well studied, the role of other Akt isoforms—highly similar, related proteins—was unknown. Sun Sik Bae at Pusan National University School of Medicine, Yangsan, South Korea, and coworkers investigated how Akt1 and Akt2 influence angiogenesis in the retinas of mice. They found that despite having very similar sequences, Akt2 had almost no effect but Akt1 was crucial for vascular stability. Akt1 boosted levels of the blood vessel stabilization protein Ang1 and suppressed Ang2. Mice lacking Akt1 eventually showed blood vessel leakage. These results illuminate the mechanisms underlying blood vessel formation.

## Introduction

The phosphatidylinositol 3-kinase (PI3K)/Akt pathway plays a crucial role in angiogenesis involving cell proliferation, migration, and survival^1^. The serine/threonine kinase Akt consists of three mammalian isoforms, namely, Akt1, Akt2, and Akt3 that share more than 80% homology in amino acid sequence^2^. Despite the high sequence homology, each Akt isoform has distinctive physiological functions. For example, the global deletion of Akt1 results in growth retardation caused by a defect in placental development^3^. Mice lacking Akt2 show normal growth but eventually develop insulin resistance and type 2 diabetes-like syndrome^4^. Akt3 knockout mice only showed reduced brain size and weight^5^. Although the role of the endothelial Akt1 isoform has widely been studied in angiogenesis^1,6^, the role of Akt and its isoform specificity in vascular smooth muscle cells (VSMCs) is relatively unknown.

Angiogenesis is the process by which new blood vessels are formed from preexisting vessels. Angiogenesis plays a crucial role in physiological conditions, including wound healing, tissue remodeling, and embryonic development, as well as in pathological conditions, such as tumor angiogenesis, retinopathy of prematurity, and diabetic retinopathy^6,7^. The process of angiogenesis involves the proliferation and migration of endothelial cells (ECs) and stabilization by the recruitment of mural cells, such as pericytes and VSMCs^8^. The interactions between ECs and VSMCs involve various ligand–receptor axes that control angiogenesis through autocrine and paracrine pathways. Vascular endothelial growth factor A (VEGFA), which binds to the VEGF receptors expressed on vascular ECs, is a key factor for endothelial cell proliferation, migration, and vascular permeability^9^. Platelet-derived growth factor (PDGF) secreted from endothelial cells induces VSMC proliferation, mural cell fate, and VSMC recruitment to the endothelium^10^. In addition, the knockout of PDGF or PDGFRβ results in a deficiency of mural cells, which leads to vascular leakage and perinatal lethality^11^. Although paracrine signaling from the endothelium to mural cells has been studied extensively, signaling from mural cells to the endothelium is poorly understood.

Angiopoietin 1 (Ang1) is a ligand of Tie2, which is strongly expressed by ECs. Ang1 is constitutively expressed by pericytes and VSMCs and plays a critical role in vessel maturation and stability^12^. In contrast, Ang2, which is an antagonistic ligand for the Tie2 receptor, acts as a negative regulator of the Tie2/Ang1 signaling cascade during angiogenesis^13^. For example, Ang2 destabilizes early vessels, and Ang1 matures the vessels to induce functional neovasculature^14^. Both Ang1 and Ang2 are expressed in ECs and exert their function in an autocrine manner^15–17^. On the other hand, recent evidence suggests that Ang1 and Ang2 are expressed in ECs, pericytes, and VSMCs, suggesting a paracrine role in the vasculature^18,19^.

The Notch signaling pathway is an additional ligand–receptor axis that regulates cell–cell communication. Mammals have four Notch receptors, Notch 1 to 4, and five Notch ligands, Dll1, Dll3, Dll4, Jagged (Jag)1, and Jag2^20^. After ligands bind to the Notch receptor, proteolytic cleavage releases the Notch intracellular domain (NICD), which translocates to the nucleus and binds with the transcription factor CSL (CBF-1, Su(H), and Lag-1) and coactivator Mastermind-like (MAML) to lead to downstream gene expression. Among the Notch isoforms, Notch3 is predominantly expressed in VSMCs and is required for arterial differentiation and VSMC maturation^21^. In addition, it has been reported that Notch3 is important in the proliferation and maturation of both pericytes and VSMCs^22,23^. On the other hand, the downstream genes of Notch3 and their roles in the vasculature are unknown.

The Hippo pathway is essential for controlling organ size and tumorigenesis^24^ and is activated by diverse stimuli, including cell polarity, cell density, and mechanical stress, to suppress Yes-associated protein (YAP) and its paralog transcription activator with PDZ binding motif (TAZ) transcriptional activity^25^. YAP is a transcriptional coactivator and is phosphorylated by LATS1/2^26,27^. LATS-dependent phosphorylation of YAP interacts with 14-3-3 and inhibits YAP nuclear localization, which leads to β-TrCP-mediated proteasomal degradation^28,29^. Recently, it has been reported that PI3K induces YAP nuclear localization by inhibiting LATS activity^30,31^, and YAP is a critical mediator of PI3K-induced mammary tumorigenesis^32^. In addition, the specific knockout of YAP in VSMCs in mice resulted in the aberrant development of large arteries and perinatal lethality^33^. On the other hand, the precise molecular target of YAP in VSMCs remains to be elucidated.

In the present study, we investigated the role of Akt1 in VSMCs during retinal angiogenesis. In particular, we provide novel evidence that Akt1 in VSMCs regulates endothelial cell functions by modulating Ang1 and Ang2 expression through Notch3 and YAP, respectively.

## Materials and methods

### Animals

Akt1^WT^ (B6.129S4(FVB)-*Akt1*^*tm2.2Mbb*^/J) and Akt2^WT^ (B6.129-*Akt2*^*tm1.2Mbb*^/J) mice were generously provided by Dr. Morris Birnbaum (University of Pennsylvania, USA). PTEN^WT^ mice were provided by Dr. Jin Woo Kim (KAIST, Daejeon, Korea). Tie2-Cre (B6.Cg-Tg(*Tek-Cre*)1Ywa/J) mice were purchased from The Jackson Laboratory (Bar Harbor, ME, USA). SM22α-Cre (B6.Cg-Tg(*Tagln-Cre*)1Her/J) mice were kindly provided by Dr. Pann-Ghill Suh (UNIST, Ulsan, Korea). Akt1^WT^ mice, Akt2^WT^ mice, or PTEN^WT^ mice were crossed with Tie2-Cre or SM22α-Cre mice to generate conditional knockout mice. Three-week-old male Sprague–Dawley rats and C57BL/6 (wild type) mice were purchased from Koatech Inc. (Pyeongtaek, Korea). The C57BLKS/J-*db/db* mice were obtained from Central Lab. Animal, Inc. (Seoul, Korea). The animals were housed under specific pathogen-free (SPF) facilities and fed a standard diet (Purina #38057, Cargill Agri Purina, Seoul, Korea) with free access to food and water.

### Materials

All endothelial cell culture media (EGM-2) were obtained from Lonza, Inc. (Walkersville, MD, USA). Dulbecco’s modified Eagle’s medium (DMEM), fetal bovine serum (FBS), trypsin-EDTA, and penicillin/streptomycin (antibiotics) were purchased from HyClone Laboratories Inc. (Logan, UT, USA). Antibodies against Akt1, pan-Akt, phospho-Akt (Ser473), Notch1, YAP, phospho-YAP (Ser127), phospho-Histone H3 (Ser10), and Histone H3 were purchased from Cell Signaling Technology (Boston, MA, USA). Anti-Akt1, anti-Akt2, and anti-NG2 antibodies were purchased from Millipore Bioscience (Temecula, CA, USA). Anti-actin antibody was obtained from MP Biomedicals (Aurora, OH, USA). Anti-YAP and anti-GAPDH were purchased from Santa Cruz Biotechnology (California, CA, USA). Antibodies against Ang1 and SM22α were purchased from Abcam (Cambridge, UK). Antibodies against Notch3 and Ang2 were obtained from Proteintech Group, Inc. (Chicago, IL, USA). Anti-SMA and anti-Flag antibodies were purchased from Sigma–Aldrich (St. Louis, MO, USA). GSL isolectin B4 (IB4) was obtained from Vector Laboratories (Burlingame, CA, USA). Alexa Fluor 405-, 488- and 546-conjugated streptavidin, Alexa Fluor 488-conjugated goat anti-mouse, Alexa Fluor 488-conjugated goat anti-rabbit, Cy3-conjugated goat anti-rabbit, Alexa Fluor 555-conjugated donkey anti-rabbit, Alexa Fluor 488-donkey anti-goat secondary antibodies and 4′,6-diamidino-2-phenylindole (DAPI) were purchased from Molecular Probes, Inc. (Carlsbad, CA, USA). VEGF was purchased from KOMA Biotech (Seoul, Korea). Recombinant COMP-Ang1 was purchased from Enzo Life Sciences, Inc. (Farmingdale, NY, USA). Recombinant human Ang2 was obtained from ReliaTech (Wolfenbüttel, Germany). PDGF-BB and FITC-dextran (2000 kDa) were purchased from Sigma–Aldrich (St. Louis, MO, USA). IRDye700- and IRDye800-conjugated rabbit and mouse secondary antibodies were obtained from Li-COR Bioscience (Lincoln, NE, USA).

### Cell isolation and cell culture

VSMCs were isolated from 3-week-old Sprague–Dawley rats or 8-week-old mice using a tissue explanting method, as described previously^34^. The thoracic aorta was isolated, and the surrounding fat and connective tissues were discarded. The vessels were cut longitudinally, and the lumen side was scraped with a razor blade to remove the intima. The vessels were fragmented into 3–5 mm lengths and explanted lumen side down on collagen-coated culture dishes. After seven days (rats) or fourteen days (mice) of explanting, tissue fragments were discarded, and sprouted VSMCs were collected and used (passages 2–3) for the experiments.

### Western blotting

The aortas were isolated from *Akt1*^*f/f*^ and *Akt1*^*f/f*^*-SM22α-Cre* mice and homogenized. The aortas or cells were lysed in 20 mM Tris-HCl, pH 7.4, 1 mM EGTA/EDTA, 1% Triton X-100, 1 mM Na_3_VO_4_, 10% glycerol, 1 μg/ml leupeptin and 1 μg/ml aprotinin. After centrifugation, the aorta or cell lysates were subjected to sodium dodecyl sulfate-polyacrylamide gel electrophoresis on 10% polyacrylamide gels and transferred to nitrocellulose membranes, which were immunoblotted using the indicated primary antibodies and IRDye-conjugated secondary antibodies (Li-COR Biosciences, Lincoln, NE, USA). The western blots were developed using the Odyssey system (Li-COR Biosciences).

### Immunocytochemistry and immunohistochemistry

For immunocytochemistry, VSMCs were grown in six-well plates on collagen-coated coverslips. Cells were washed with ice-cold PBS and fixed with 4% paraformaldehyde for 15 min. The cells were permeabilized with 0.2% Triton X-100 and incubated with the indicated primary antibodies for 2 h. They were then treated with Cy3- or Alexa Fluor 488-conjugated secondary antibodies for 1 h. The samples were mounted with an anti-fading reagent (2% *n*-propylgalate in 80% glycerol/PBS solution), and images were obtained using a confocal microscope (K1-Fluo, Nanoscope Systems, Daejeon, Korea). For immunohistochemistry, the mice were perfused with PBS, and the isolated aortas were fixed with 4% paraformaldehyde at 4 °C overnight and embedded in paraffin. Five-micrometer sections of each block were stained with the indicated primary antibody at 4 °C overnight and incubated with Cy3- or Alexa Fluor 488-conjugated secondary antibodies for 2 h. The images were visualized using a confocal microscope (K1-Fluo, Nanoscope Systems, Daejeon, Korea) and quantified using ImageJ (National Institutes of Health).

### Aortic sprouting assay

Growth factor-reduced Matrigel (BD Bioscience, San Jose, CA, USA) was thawed on ice, and 150 μl of Matrigel was plated into precooled 48-well plates. Thoracic aortas were dissected from 6- to 7-week-old Akt1^WT^, Akt1^∆SMC^, Akt2^WT^, and Akt2^∆SMC^ mice, and the surrounding fat and connective tissues were discarded. The aortas were cut into 0.8-mm-long aortic rings, embedded in Matrigel-coated wells, and incubated for 30 min at 37 °C to allow for polymerization. The aortic rings from Akt1^WT^ mice were stimulated either with Ang1 neutralizing antibody or with recombinant Ang2 or Ang1 neutralizing antibody together with recombinant Ang2 in the presence of EGM-2 medium (Lonza, Inc.). For the Akt1^∆SMC^ mice, the aortic rings were stimulated either with Ang2 neutralizing antibody or with recombinant COMP-Ang1 or Ang2 neutralizing antibody together with recombinant COMP-Ang1 in the presence of EGM-2 medium. The medium was changed every other day, and sprouting aortic rings were quantified at 8 days after embedding. The images were obtained using a fluorescence microscope at ×5 magnification (Axiovert200, Carl Zeiss, Jena, Germany). Quantification of the sprouting area is presented as a percentage of the control using ImageJ (National Institutes of Health).

### Whole-mount staining of the retina

Eyes were isolated from the indicated mice and fixed in 4% paraformaldehyde for 12 h at 4 °C. The cornea, sclera, lens, and hyaloid vessels were removed, and the retinas were blocked and permeabilized in blocking buffer (1% BSA and 0.3% Triton X-100 in PBS) for 12 h at 4 °C. For immunostaining, IB4 was diluted in PBlec solution (1% Triton X-100, 1 mM CaCl_2_, 1 mM MnCl_2_, and 1 mM MgCl_2_ in PBS, pH 6.8), and other primary antibodies were incubated in retinal blocking buffer overnight at 4 °C. The secondary antibodies were diluted in retinal blocking buffer and incubated for 2 h at room temperature. Retinas were flat-mounted with an anti-fading reagent (2% *n*-propylgalate in 80% glycerol/PBS solution), and images were obtained using a confocal microscope (K1-Fluo, Nanoscope Systems, Daejeon, Korea). Retinal angiogenesis was analyzed by measuring the percentage of angiogenic area per total area, sprouting vessel distance from the optic nerve, number of tip cells per field, and filopodia lengths. The proliferation of retinal ECs and mural cells was quantified by counting the IB4- and pH3-positive cells (white) or pH3-positive cells (purple), located next to IB4 staining, respectively. The pericyte and VSMC coverage was quantified from the fluorescence intensity using ImageJ (National Institutes of Health).

### Short-hairpin RNA and constructs

To silence Akt1, Akt2, Notch3 and YAP, shAkt1 (5′-CGA GTT TGA GTA CCT GAA GCT-3′), shAkt2 (5′-CGA CCC AAC ACC TTT GTC ATA-3′), shNotch3 (5′-GGC ACA CAT TGC CAA TAT A-3′), and shYAP (5′-GCC ATG AAC CAG AGG ATC A-3′) oligonucleotides with an *AgeI* site at the 5′-end and an *EcoRI* site at the 3′ end were designed, and sense and antisense oligonucleotides were synthesized (XENOTECH, Daejeon, Korea). Both complementary oligonucleotides were mixed, heated to 98 °C for 5 min, and cooled to room temperature. The annealed nucleotides were subcloned into the *AgeI/EcoRI* sites of the pLKO.1 lentiviral vector.

### Lentiviral knockdown

For gene silencing, HEK293-FT packaging cells (Invitrogen, Carlsbad, CA, USA) were grown to ~70% confluence in 100 mm cell culture dishes. The cells were triple transfected with 20 μg of pLKO.1 lentiviral vector (control) or vector containing shAkt1, shAkt2, shNotch3, or shYAP; 5 μg of Δ8.9; and 5 μg of pVSV-G using the calcium phosphate method. The medium was replaced with fresh medium 8 h after transfection. Lentiviral supernatants were harvested 24 h and 48 h after transfection and passed through 0.45 μm filters. Cell-free viral culture supernatants were used to infect contractile VSMCs in the presence of 8 μg/ml polybrene (Sigma-Aldrich, St. Louis, MO, USA). After puromycin selection (10 μg/ml) for 2 days, at least 95% of cells survived and were used for the experiments.

### Retroviral gene expression

pIRES-Flag-Notch3-IC was provided by Dr. Hee Sae Park (Chonnam National University, Gwangju, Korea). Notch3-IC cDNA was subcloned into the *BamHI/EcoRI* site of the pMIGR2 vector. pCMV2-Flag-YAP2 was provided by Dr. Eek-Hoon Jho (University of Seoul, Seoul, Korea). YAP2 cDNA was subcloned into the *EcoRI* site of the pMIGR2 vector. Generation of retroviral particles for the expression of genes and their infection were performed essentially, as described previously^35^.

### Analysis of mRNA expression

The expression of Ang1, Ang2, YAP, and Notch target gene mRNA was measured by reverse transcription polymerase chain reaction (RT–PCR) and real-time quantitative PCR (Q-PCR) analysis after isolating the total RNA using TRIzol reagent, as described in the manufacturer’s protocol (Invitrogen, Grand Island, NY, USA). One hundred fifty nanograms of the total RNA were reverse transcribed into cDNA using M-MLV reverse transcriptase (Promega Biotech, Madison, WI, USA); the cDNA was then amplified by PCR using the specific primers for mouse Akt1 (forward, 5′-GCC CAA CAC CTT TAT CAT CC-3′; reverse, 5′-GTC CAT CGT CTC TTC TTC CTG-3′), mouse Ang1 (forward, 5′-AGG CTT GGT TTC TCG TCA GA-3′; reverse, 5′-TCT GCA CAG TCT CGA AAT GG-3′), mouse Ang2 (forward, 5′-GAA CCA GAC AGC AGC ACA AA-3′; reverse, 5′-AGT TGG GGA AGG TCA GTG TG-3′), mouse GAPDH (forward, 5′-TGT GAA CGG ATT TGG CCG TA-3′; reverse, 5′-ACT GTG CCG TTG AAT TTG CC-3′), rat Ang1 (forward, 5′-GGA GTC CAG AAA ACG GAG GG-3′; reverse, 5′-TTT GCA GAG CGT TGG TGT TG-3′), rat Ang2 (forward, 5′-GGA CCC TGC AGC TAC ACA TT-3′; reverse, 5′-CGG CGT TAG ACA TGT AGG GG-3′), rat Notch3 (forward, 5′-TAC TGG ACC TCG CTG TGA GA-3′; reverse, 5′-TGG CCA ATT CGG TCA AGA CA-3′), rat Hes1 (forward, 5′-GCT TCA GCG AGT GCA TGA AC-3′; reverse, 5′-CGG TGT TAA CGC CCT CAC A-3′), rat Hes5 (forward, 5′-GCA CCA GCC CAA CTC CAA-3′; reverse, 5′-GGC GAA GGC TTT GCT GTG-3′), rat Hey1 (forward, 5′-CAC TGC AGG AGG GAA AGG TTA T-3′; reverse, 5′-CCC CAA ACT CCG ATA GTC CAT-3′), rat Hey2 (forward, 5′-AGA CGA CCT CTG AAA GCG AC-3′; reverse, 5′-TTC GAT CCC GAC GCC TTT TT-3′), rat YAP (forward, 5′-CTT CTG GTC AGA GAT ACT TCT-3′; reverse, 5′-TCT GGT TCA TGG CAA AAC GAG-3′) and rat GAPDH (forward, 5′-AGG TCG GTG TGA ACG GAT TT-3′; reverse, 5′-CCA CTT TGT CAC AAG AGA AGG C-3′). Equal amounts of RT–PCR products were separated on a 2% agarose gel and stained with ethidium bromide. Q-PCR data were analyzed using Roche Light Cycler 96 software (Roche Diagnostics) and the comparative *C*_t_ method^36^. Calibration was based on the expression of GAPDH.

### Luciferase reporter assay

To assess promoter activity, the dual-luciferase reporter assay system was employed. The TEAD luciferase reporter 8xGTIIC-luciferase plasmid was a gift from Dr. Eek-Hoon Jo (University of Seoul, Korea). VSMCs were seeded in 12-well plates and cotransfected with the luciferase reporter constructs and Renilla luciferase plasmids using Lipofectamine 2000 (Invitrogen, Carlsbad, CA, USA). Each well contained 0.8 μg of the luciferase reporter plasmid and 80 ng of Renilla luciferase plasmid. The medium was replaced with fresh medium 7 h posttransfection. The cells were lysed and assayed for luciferase activity 24 h after transfection. Twenty microliters of protein extracts were analyzed using a GloMax^TM^ 20/20 luminometer (Promega, WI, USA). The Ang2 promoter was a gift from Young-Guen Kwon (Yonsei University, Korea). VSMCs were transfected with the Ang2 promoter reporter and were incubated for 48 h. The cell culture medium was collected, and Gaussia luciferase activity was measured according to the manufacturer’s protocol (GeneCopoeia Inc.).

### Corneal angiogenesis assay

The corneal micropocket assay was performed as described^37,38^. Seven-week-old Akt1^WT^ and Akt1^∆SMC^ mice were anesthetized with chloral hydrate (450 mg/kg, i.p.). After 10 min, alcaine was dropped into the eye. A corneal micropocket was created with a modified von Graef knife and MVR knife in both eyes. A micropellet of sucralfate (Sigma-Aldrich) coated with hydron polymer (Sigma–Aldrich) containing 200 ng of VEGF was implanted into each corneal pocket. The pellet was positioned approximately 1 mm from the corneal lymbus. Seven days later, the mice were anesthetized with 1–2% inhaled isoflurane, and the eyes were captured using a digital camera. For staining, eyes were fixed with 4% paraformaldehyde for 12 h at 4 °C. The primary antibody was incubated in blocking buffer (3% BSA in PBS-Tween-20) overnight at 4 °C. The secondary antibody was diluted in blocking buffer and incubated for 2 h at room temperature. Corneas were flat-mounted using an anti-fading reagent, and images were obtained using a confocal microscope (K1-Fluo, Nanoscope Systems, Daejeon, Korea). Sprouting was quantified by measuring the VEGF-induced vessel sprouting length using ImageJ (National Institutes of Health).

### Tumor angiogenesis assay

B16-BL6 melanoma cells (4 × 10^5^) were injected subcutaneously into the backs of 6-week-old Akt1^WT^, Akt1^∆SMC^, Akt2^WT^, and Akt2^∆SMC^ mice. Two weeks after injection, tumor weights and volumes were measured using a scale. The images were captured using a digital camera.

### Diabetic retinopathy and vascular permeability assay

Diabetes was induced in mice by a daily intraperitoneal injection of STZ (50 mg/kg in 0.1 M sodium citrate buffer, pH 4.5, Sigma–Aldrich) for five consecutive days in 8-week-old male Akt1^WT^ and Akt1^∆SMC^ mice. The blood glucose levels were measured using a blood glucose test meter (Accu-Check Active; Roche Diagnostics) by tail vein puncture blood sampling. Blood glucose levels were measured every week to confirm hyperglycemia. After 7 weeks of injection, the mice were anesthetized with an intraperitoneal injection of ketamine (80 mg/kg) and xylazine (10 mg/kg), and 200 μl of FITC-dextran (2000 kDa, 50 mg/ml in sterile PBS) was injected into the left ventricle of the mice. Five minutes after the injection, the mice were euthanized in a CO_2_ chamber, and the eyes were isolated and fixed with 4% paraformaldehyde for 12 h at 4 °C. Retinal images were obtained using a confocal microscope (K1-Fluo, Nanoscope Systems, Daejeon, Korea). Vascular leakage was quantified using MetaMorph software (Molecular Devices, Sunnyvale, CA).

### Statistical analysis

The data were plotted and analyzed using GraphPad Prism. Unpaired Student’s *t* test (two tails) was used to determine the significance of the intergroup differences. Multiple sets of data were analyzed by analysis of variance (one-way ANOVA) and Tukey’s multiple comparison test. The results are expressed as the means ± SEM, and *P* values less than 0.05 were considered significant.

## Results

### Expression of Akt1 in VSMCs as well as in ECs is required for retinal angiogenesis

**Fig. 1:**
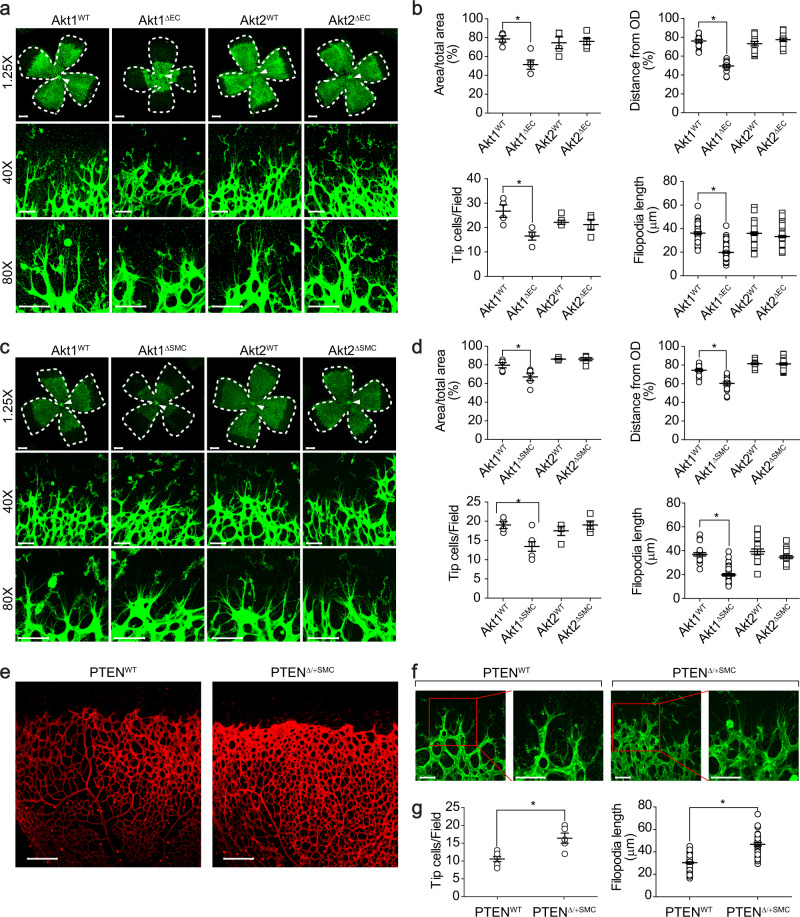
Angiogenesis is regulated by Akt1 in VSMCs. **a** Retinas isolated from Akt1^WT^, Akt1^∆EC^, Akt2^WT^, and Akt2^∆EC^ mice at P6 were stained with IB4 (green). The images were obtained using a confocal microscope at ×1.25 (area), ×40 (tip cells), and ×80 (filopodia) magnification. Bar, 800 μm (×1.25) and 50 μm (×40/80). **b** Quantification of the angiogenic area (*n* = 4–6), sprouting distance from the optic nerve (indicated by white arrows, *n* = 14), tip cell numbers (*n* = 4–6), and filopodia lengths (*n* = 47). *P < 0.05, one-way ANOVA followed by Tukey’s multiple comparison test. **c** Retinas were stained with IB4 (green). Bar, 800 μm (×1.25), and 50 μm (×40/80). **d** Quantification of the angiogenic area (*n* = 4–6), sprouting distance from the optic nerve (*n* = 16), tip cell numbers (*n* = 4–7) and filopodia lengths (*n* = 18). *P < 0.05, one-way ANOVA followed by Tukey’s multiple comparison test. **e** Retinas were isolated from PTEN^WT^ and PTEN^∆/+SMC^ mice at P6 and stained with IB4 (red) (*n* = 5). Bar, 200 μm. **f**, **g** Retinas were stained with IB4 (green), and tip cell numbers (*n* = 5–7) and the filopodia length (*n* = 44) were quantified with Student’s *t* test. Bar, 50 μm. *P < 0.05. The data are presented as the mean ± SEM.

To investigate the role of Akt in retinal angiogenesis, mice were generated with either EC-specific or VSMC-specific deletion of the Akt isoforms (Supplementary Fig. 1a–d). The outgrowth of the superficial retinal vascular plexus was significantly delayed in EC-specific Akt1-deficient mice (Fig. 1a, b). In addition, the angiogenic area and sprouting distance from the optic nerve were significantly impaired, and tip cell numbers and filopodia lengths were significantly reduced in the retinas of EC-specific Akt1-deficient (Akt1^∆EC^) mice. On the other hand, Akt2^∆EC^ mice showed normal angiogenesis compared to that of the wild type mice. Next, we investigated the effects of deleting the Akt isoform from VSMCs. Both Akt1 and Akt2 were selectively disrupted in VSMCs (Supplementary Fig. 1f, g). Mice with Akt1^∆SMC^ showed similar results to Akt1^∆EC^ mice (Fig. 1c, d). However, mice with Akt2^∆SMC^ had no effect on retinal angiogenesis. To further confirm the effect of Akt1 in VSMCs, a mouse model with enhanced Akt activity was generated by disrupting PTEN, which is a negative regulator of Akt, in VSMCs (Supplementary Fig. 1e). Enhanced tip cell numbers and vessel density were observed in mice with PTEN^∆/+SMC^ (Fig. 1e–g).

### Expression of Akt1 in VSMCs is essential for corneal and tumor angiogenesis

**Fig. 2:**
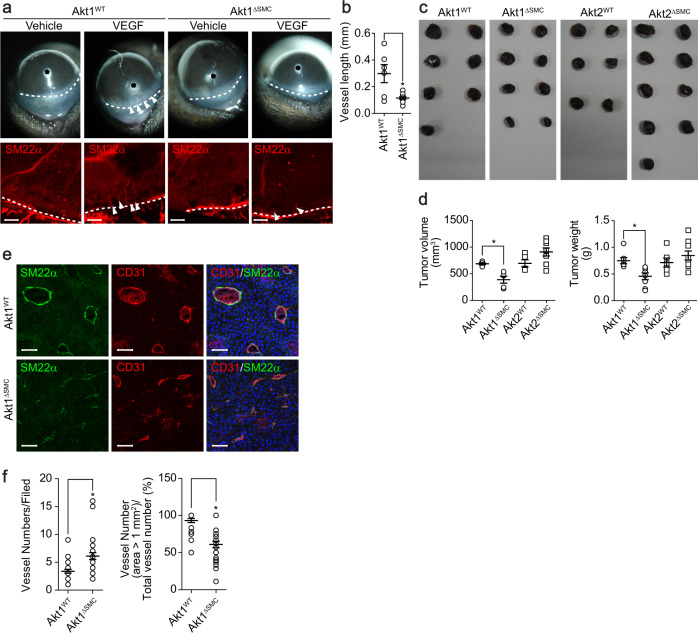
Inhibition of corneal and tumor angiogenesis in VSMC-specific Akt1-deficient mice. **a** A corneal micropocket (indicated by the black asterisks) was created in Akt1^WT^ and Akt1^∆SMC^ mice, and a micropellet containing 200 ng of VEGF was implanted into each corneal pocket. The eyes were imaged using a digital camera and stained with SM22α (red) antibody. White arrows indicate newly formed blood vessels. Bar, 200 μm. **b** Quantification of the VEGF-induced vessel length (*n* = 6). *P < 0.05, Student’s *t* test. **c** Melanoma cells were injected into Akt1^WT^, Akt1^∆SMC^, Akt2^WT^, and Akt2^∆SMC^ mice. Two weeks after injection, tumors were isolated, and images were captured using a digital camera. **d** Tumor volumes (*n* = 5–8) and weights (*n* = 6–9) were measured using a scale. *P < 0.05, one-way ANOVA followed by Tukey’s multiple comparison test. **e** Tumors were stained with SM22α (green) and CD31 (red). Bar, 100 μm. **f** Quantification of vessel numbers (*n* = 25). *P < 0.05, Student’s *t* test. The data are presented as the mean ± SEM.

The role of Akt in VSMCs in corneal angiogenesis was next explored. Sucralfate coated with a slow-release hydron polymer containing 200 ng of VEGF was surgically implanted into the corneas of VSMC-specific Akt isoform-deficient mice. The implantation of VEGF beads into the corneas of Akt1^WT^ mice induced angiogenesis from the corneal limbus to the beads. However, VEGF-induced microvessel sprouting was significantly inhibited in Akt1^∆SMC^ mice (Fig. 2a, b). Tumor angiogenesis was examined by subcutaneously injecting B16BL6 melanomas into Akt1^∆SMC^ and Akt2^∆SMC^ mice to further determine the role of Akt1 in VSMCs. Tumor volumes and weights were significantly reduced in Akt1^∆SMC^ mice. However, Akt2^∆SMC^ mice showed no effect (Fig. 2c, d). In addition, different vessel structures, numbers, and sizes were observed in Akt1^∆SMC^ mice. In particular, vessel numbers were increased, whereas the number of vessels larger than 1 mm^2^ was reduced in Akt1^∆SMC^ mice (Fig. 2e, f).

### Akt1 in VSMCs regulates EC proliferation as well as mural cell coverage

**Fig. 3:**
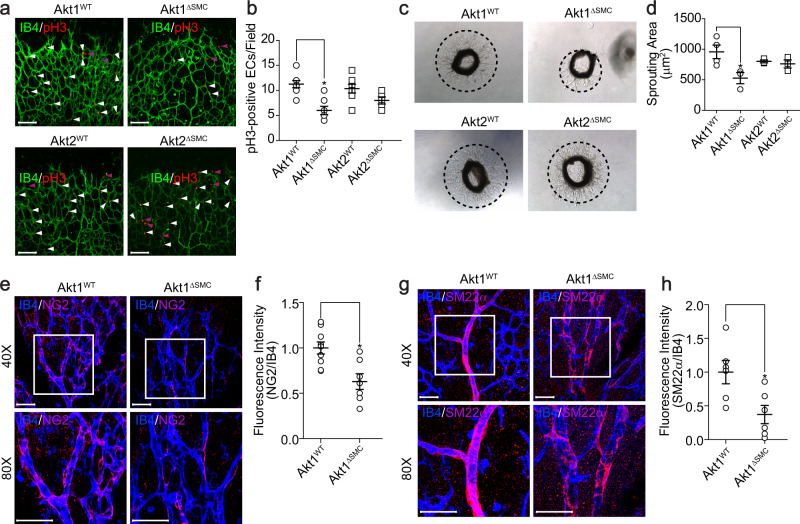
Defects in EC proliferation and mural cell coverage in Akt1∆SMC mice. **a** Retinas were isolated from Akt1^WT^, Akt1^∆SMC^, Akt2^WT^, and Akt2^∆SMC^ mice at P6 and stained with IB4 (green) and pH3 (red). White arrowheads indicate IB4- and pH3-positive ECs, and purple arrowheads indicate pH3-positive mural cells. Bar, 100 μm. **b** Quantification of the number of pH3-positive ECs (*n* = 5–8). *P < 0.05, one-way ANOVA followed by Tukey’s multiple comparison test. **c** Aortas were embedded in growth factor-reduced Matrigel-coated plates in the presence of EGM-2. After 7 days, bright field images were captured under an optical microscope at ×5 magnification. **d** Quantification of the sprouting area (*n* = 3–4). *P < 0.05, one-way ANOVA followed by Tukey’s multiple comparison test. **e**, **g** Retinas were isolated from Akt1^WT^ and Akt1^∆SMC^ mice at P4 and stained with IB4 (blue), NG2, or SM22α (red). The images were visualized under a confocal microscope at ×40 (top) and ×80 (bottom) magnification. Bar, 50 μm. **f**, **h** The fluorescence intensity of NG2/IB4 (*n* = 7–9) and SM22α/IB4 (*n* = 6) was quantified with Student’s *t* test. *P < 0.05. The data are presented as the mean ± SEM.

Since the data showed that Akt1 in VSMCs regulated the physiological responses of ECs, such as the number of tip cells and the filopodia length in tip cells (Fig. 1c, d), this study next examined the effect of Akt1^∆SMC^ or Akt2^∆SMC^ on the proliferation of ECs. The proliferation of ECs from Akt1^∆SMC^ mice was significantly reduced, whereas Akt2^∆SMC^ mice showed normal EC proliferation compared to the wild type (Fig. 3a, b). To further confirm VSMC-dependent proliferation of ECs ex vivo, an aortic sprouting assay was performed. EC sprouting was markedly reduced in the aortic rings from Akt1^∆SMC^ mice, whereas EC sprouting from Akt2^∆SMC^ mice was similar to that from wild-type mice (Fig. 3c, d). Since it is possible that mural cell recruitment to and coverage of the endothelium could affect EC proliferation, we examined pericyte recruitment and VSMC coverage. The recruitment of pericytes (NG2) to and VSMC coverage (SM22α) of the endothelium were significantly reduced in Akt1^∆SMC^ mice (Fig. 3e–h).

### Akt1 regulates Ang1 and Ang2 expression in VSMCs

**Fig. 4:**
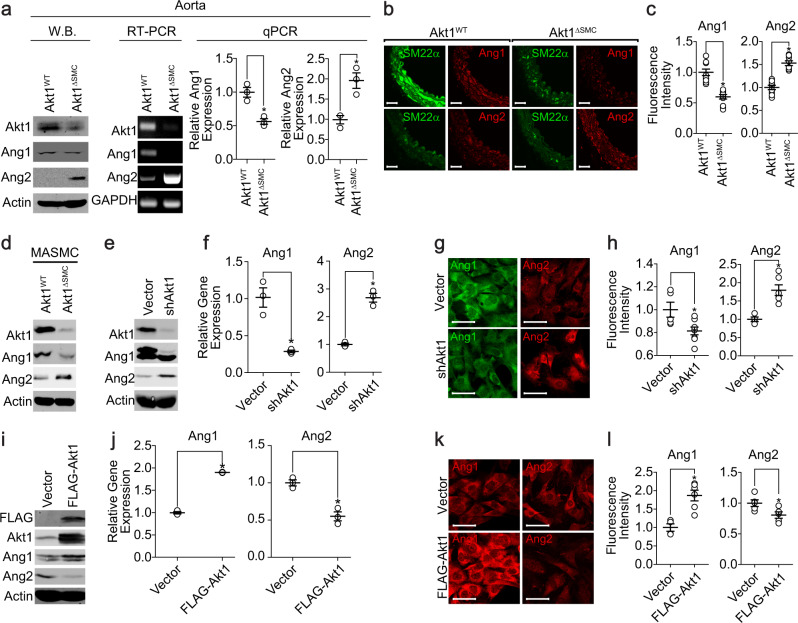
Regulation of Ang1 and Ang2 expression by Akt1 in VSMCs. **a** Aortas were isolated from Akt1^WT^ and Akt1^∆SMC^ mice, and the expression of Akt1, Ang1, and Ang2 was verified by western blot, RT–PCR, and qPCR (*n* = 3) analysis. *P < 0.05, Student’s *t* test. **b** Aortas were stained with SM22α (green), Ang1 (red), or Ang2 (red). Images were visualized under a confocal microscope at ×40 magnification. Bar, 50 μm. **c** The fluorescence intensities of Ang1 (*n* = 11) and Ang2 (*n* = 8) were quantified with Student’s *t* test. *P < 0.05. **d** MASMCs were isolated from Akt1^WT^ and Akt1^∆SMC^ mice, and gene expression was assessed by western blot analysis. Akt1 expression was silenced in VSMCs, and the levels of Ang1 and Ang2 expression were verified by western blotting (**e**), qPCR (**f**), and immunocytochemistry **g** analysis (*n* = 3). The images were visualized under a confocal microscope at ×40 (Ang2) and ×80 (Ang1) magnification. Bar, 50 μm. *P < 0.05, Student’s *t* test. **h** The fluorescence intensities of Ang1 (*n* = 5–8) and Ang2 (*n* = 4–6) were quantified with Student’s *t* test. *P < 0.05. Akt1 was ectopically expressed in VSMCs, and the expression levels of Akt1, Ang1, and Ang2 were assessed by western blotting (**i**), qPCR (**j**), and immunocytochemistry **k** analysis (*n* = 3). Bar, 50 μm. *P < 0.05, Student’s *t* test. **l** The fluorescence intensities of Ang1 (*n* = 4–6) and Ang2 (*n* = 5) were quantified with Student’s *t* test. *P < 0.05. The data are presented as the mean ± SEM.

Several angiogenic factors expressed in an Akt1-dependent manner were screened to explore the paracrine factors secreted from VSMCs. To verify the expression of Ang1 and Ang2 in vascular cell types, we analyzed a single-cell RNA-sequencing database provided on the website (http://betsholtzlab.org/VascularSingleCells/database.html)^39,40^. Single-cell RNA-sequencing analysis showed that adequate amounts of Ang1 and Ang2 were expressed in VSMCs and pericytes in both the brain and lung vasculature (Supplementary Fig. 2). The expression of Ang1 was significantly reduced in the aortas from Akt1^∆SMC^ mice, whereas the expression of Ang2 was significantly upregulated (Fig. 4a–c). In addition, a similar pattern of Ang1 and Ang2 expression was observed in the smooth muscle cells isolated from the aortas of Akt1^∆SMC^ mice (Fig. 4d). Akt1 was silenced in primary VSMCs isolated from rat aortas to confirm the effect of Akt1 on the expression of Ang1 and Ang2 in vitro. The silencing of Akt1 significantly downregulated Ang1 expression, whereas Ang2 expression was significantly upregulated (Fig. 4e–h). In contrast, ectopic overexpression of Akt1 significantly enhanced Ang1 expression but suppressed Ang2 expression (Fig. 4i–l).

### Akt1 regulates Ang1 expression through modulation of Notch3 activation in VSMCs

**Fig. 5:**
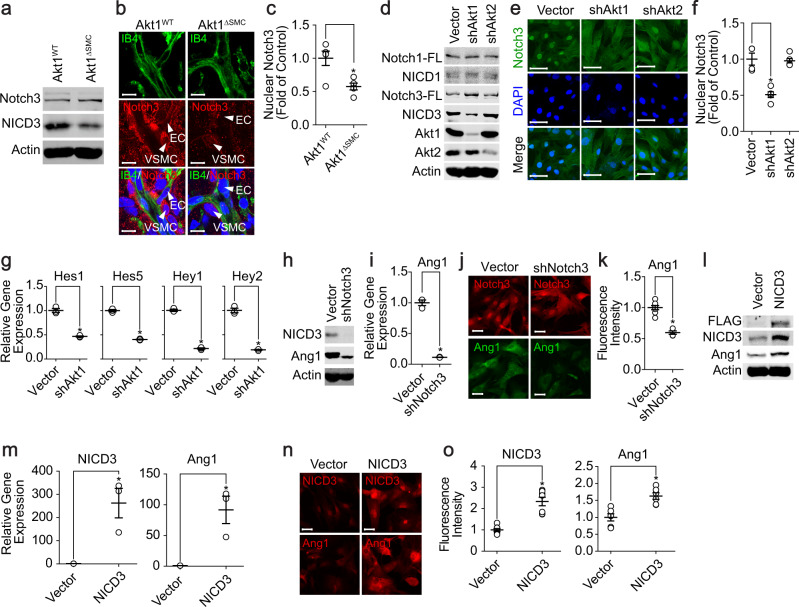
Notch3 regulates the expression of Ang1 via the Akt1 signaling pathway. **a** MASMCs were isolated from Akt1^WT^ and Akt1^∆SMC^ mice, and the activation of Notch3 was assessed by western blot analysis. **b** Retinas were stained with IB4 (green), Notch3 (red), and DAPI (blue). Bar, 10 μm. **c** The fluorescence intensity of nuclear Notch3 (*n* = 4–5) was quantified with Student’s *t* test. *P < 0.05. Akt1 or Akt2 was silenced in VSMCs, and the expression of Akt1/2 and activation of Notch1 and Notch3 were verified by western blot analysis (**d**) and immunocytochemistry (**e**). Bar, 50 μm. **f** Quantification of nuclear Notch3 expression (*n* = 5). *P < 0.05, one-way ANOVA followed by Tukey’s multiple comparison test. **g** Akt1 was silenced in VSMCs, and the expression of Notch3 target genes was verified by qPCR (*n* = 3). *P < 0.05, Student’s *t* test. **h**–**j** Notch3 was silenced in VSMCs, and the expression of Ang1 was assessed by western blotting (**h**), qPCR (**i**), and immunocytochemistry (**j**) (*n* = 3). Bar, 50 μm. *P < 0.05, Student’s *t* test. **k** The fluorescence intensity of Ang1 (*n* = 5–8) was quantified with Student’s *t* test. *P < 0.05. NICD3 was ectopically expressed in VSMCs, and Ang1 expression was verified by western blotting (**l**), qPCR (**m**), and immunocytochemistry (**n**) (*n* = 3). Bar, 50 μm. *P < 0.05, Student’s *t* test. **o** The fluorescence intensity of NICD3 (*n* = 7) and Ang1 (*n* = 6) was quantified with Student’s *t* test. *P < 0.05. The data are presented as the mean ± SEM.

Since Notch1 and Notch3 are expressed in VSMCs and Notch3 plays an important role in VSMC differentiation and maturation^21^, we examined the effect of Akt on the regulation of Notch1 and Notch3 activation in VSMCs. The activation of Notch3 was significantly reduced in the retinas from Akt1^∆SMC^ mice (Fig. 5a). In addition, the active form of Notch3 in the nucleus was significantly reduced in Akt1^∆SMC^ mice (Fig. 5b, c). Silencing Akt1 selectively blunted the activation of Notch3, and nuclear Notch3 was significantly abolished in VSMCs, whereas silencing Akt2 was ineffective (Fig. 5d–f). Furthermore, silencing Akt1 significantly blocked the expression of Notch3 target genes, such as Hes1, Hes5, Hey1, and Hey2 (Fig. 5g). Finally, silencing Notch3 significantly blocked the expression of Ang1, whereas ectopic expression of Notch3 significantly enhanced Ang1 expression (Fig. 5h–o).

### Akt1 regulates Ang2 expression through regulation of YAP in VSMCs

**Fig. 6:**
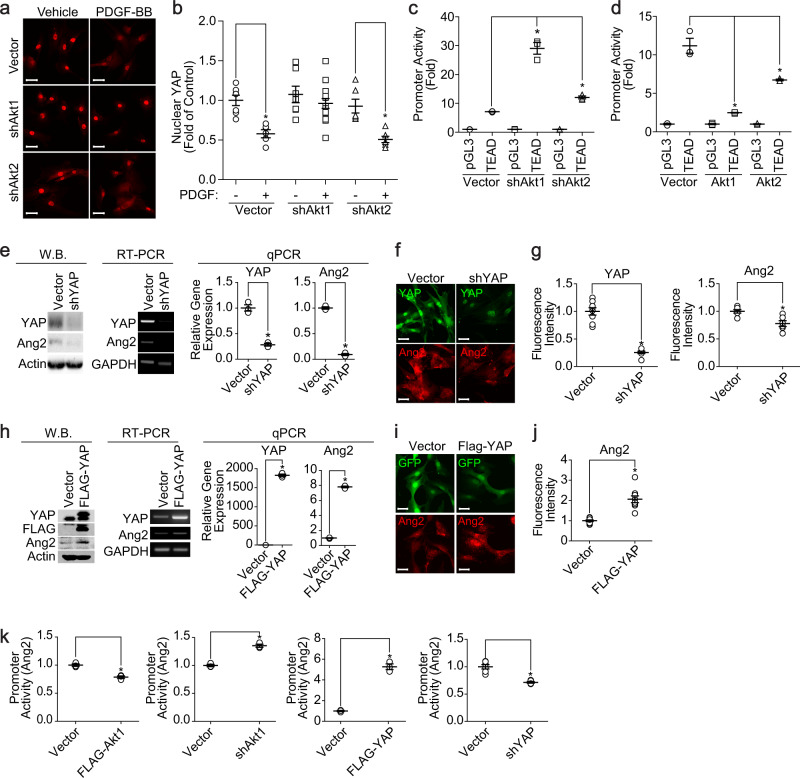
YAP regulates the expression of Ang2 via the Akt1 signaling pathway. **a** Akt1 or Akt2 was silenced in VSMCs, and cells were stimulated with PDGF-BB for 30 min and stained with YAP (red) antibody. Bar, 50 μm. **b** Quantification of nuclear YAP expression (*n* = 7 for vector, *n* = 7–15 for shAkt1, *n* = 6 for shAkt2). *P < 0.05, one-way ANOVA followed by Tukey’s multiple comparison test. The transcriptional activity of YAP was measured either after silencing (**c**) or overexpressing **d** Akt isoforms (*n* = 3). *P < 0.05, one-way ANOVA followed by Tukey’s multiple comparison test. After silencing YAP in VSMCs, the expression of Ang2 was verified by western blotting, RT–PCR, qPCR (**e**) and immunocytochemistry **f** (*n* = 3). Bar, 50 μm. *P < 0.05, Student’s *t* test. **g** Fluorescence intensity of YAP (*n* = 8) and Ang2 (*n* = 6) was quantified with Student’s *t* test. *P < 0.05. YAP was ectopically expressed in VSMCs, and the expression of Ang2 was verified by western blotting, RT–PCR, qPCR (**h**) and immunocytochemistry **i** (*n* = 3). Bar, 50 μm. *P < 0.05, Student’s *t* test. **j** The fluorescence intensity of Ang2 (*n* = 9) was quantified with Student’s *t* test. *P < 0.05. **k** After overexpression of Akt1 or YAP in VSMCs, the promoter activity of Ang2 was verified (*n* = 3). After silencing Akt1 or YAP, the promoter activity of Ang2 was verified (*n* = 3–6). *P < 0.05, Student’s *t* test. The data are presented as the mean ± SEM.

The effect of Akt1 on the regulation of YAP activation was first examined to verify the role of YAP in the Akt1-dependent regulation of Ang2 expression. PDGF-induced phosphorylation of Akt and nuclear localization of YAP were inversely correlated (Supplementary Fig. 3). In addition, PDGF suppressed the nuclear localization of YAP, which was abolished by silencing Akt1 but not by Akt2 (Fig. 6a, b). Furthermore, the transcriptional activity of YAP was significantly enhanced by silencing Akt1, which was suppressed by the overexpression of Akt1 (Fig. 6c, d). Ang2 expression was significantly abolished by silencing YAP in VSMCs (Fig. 6e–g). Ectopic expression of YAP significantly enhanced the expression of Ang2 (Fig. 6h–j). In addition, the promoter activity of Ang2 was significantly enhanced by the silencing of Akt1 or ectopic expression of YAP but significantly suppressed by the ectopic expression of Akt1 or silencing of YAP (Fig. 6k).

### The effect of Ang1 and Ang2 expression on ex vivo angiogenesis

**Fig. 7:**
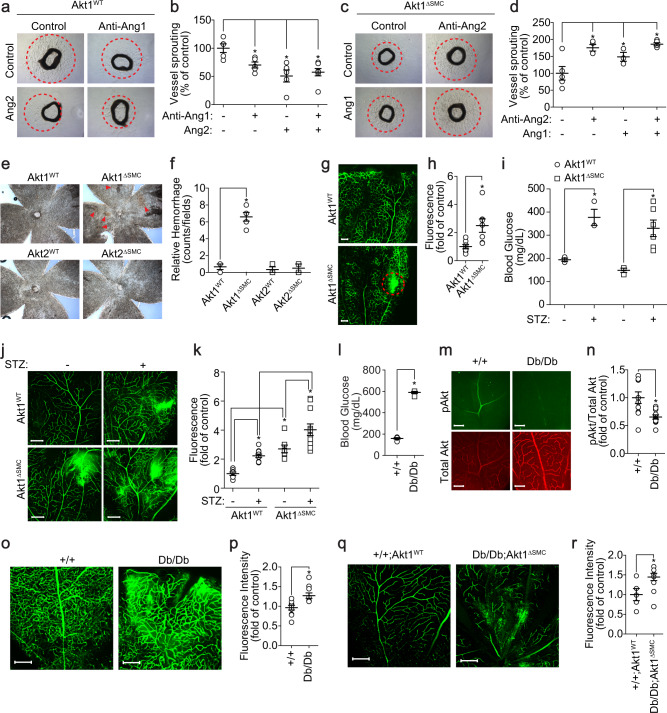
Ang1 and Ang2 regulate endothelial sprouting, and VSMC-specific disruption of Akt1 results in vessel leakage in a diabetic retinopathy mouse model. **a** Aortas were stimulated with Ang1 neutralizing antibody, recombinant Ang2, or Ang1 neutralizing antibody together with recombinant Ang2 in the presence of EGF-2 medium. After 8 days, bright field images were captured under a microscope at ×5 magnification. **b** Quantification of vessel sprouting (*n* = 5–7). *P < 0.05, one-way ANOVA followed by Tukey’s multiple comparison test. **c** Aortas were stimulated with Ang2 neutralizing antibody, recombinant COMP-Ang1 or Ang2 neutralizing antibody together with recombinant COMP-Ang1 in the presence of EGM-2 medium. After 8 days, bright field images were captured under a microscope at ×5 magnification. **d** Quantification of vessel sprouting (*n* = 3–5). *P < 0.05, one-way ANOVA followed by Tukey’s multiple comparison test. **e** Retinal vessel hemorrhage was visualized under a bright field microscope at ×5 magnification. Red arrowheads indicate hemorrhagic foci. **f** The number of hemorrhage spots (*n* = 3–5) was quantified with one-way ANOVA followed by Tukey’s multiple comparison test. *P < 0.05. **g** FITC-dextran was injected into the left ventricles, and 5 min later, retinas were isolated and images captured under a confocal microscope. Bar, 200 μm. **h** Quantification of vascular leakage (*n* = 7). *P < 0.05, Student’s *t* test. **i** Diabetes was induced in mice by a daily intraperitoneal injection of STZ (50 mg/kg) for 5 consecutive days in 8-week-old male Akt1^WT^ and Akt1^∆SMC^ mice. The blood glucose levels were measured by tail vein puncture blood sampling (*n* = 3–6). *P < 0.05, one-way ANOVA followed by Tukey’s multiple comparison test. **j** FITC-dextran was injected into the left ventricles, and 5 min later, retinas were isolated and images captured under a confocal microscope. Bar, 200 μm. **k** Quantification of vascular leakage (*n* = 7–11). *P < 0.05, one-way ANOVA followed by Tukey’s multiple comparison test. **l** Blood glucose levels were measured by tail vein puncture blood sampling (*n* = 5). *P < 0.05, Student’s *t* test. **m**, **n** Retinas were stained with pAkt (green) and total Akt (red) (*n* = 10). Bar, 200 μm. *P < 0.05, Student’s *t* test. **o**, **q** FITC-dextran was injected into the left ventricles, and 5 min later, retinas were isolated, and images were captured under a confocal microscope. Bar, 200 μm. **p** Quantification of vascular leakage (*n* = 10). *P < 0.05, Student’s *t* test. **r** Quantification of vascular leakage (*n* = 5–6). *P < 0.05, Student’s *t* test. The data are presented as the mean ± SEM.

To investigate the role of Ang1 and Ang2 expression in the activation of ECs, we examined microvessel sprouting in aortic rings from Akt1^∆SMC^ mice. EGM-2-induced vessel sprouting was markedly reduced by adding an Ang1 neutralizing antibody or recombinant Ang2 (Fig. 7a, b). Reduced microvessel sprouting in aortic rings from Akt1^∆SMC^ mice was significantly restored by adding the Ang2 neutralizing antibody or recombinant COMP-Ang1 (Fig. 7c, d).

### VSMC-specific disruption of Akt1 resulted in vessel leakage in a diabetic retinopathy mouse model

Since Akt1^∆SMC^ mice showed suppression of EC function and VSMC coverage, we verified the vascular permeability in Akt1^∆SMC^ mice. Hemorrhagic foci were observed in Akt1^∆SMC^ mice, whereas Akt2^∆SMC^ mice showed no hemorrhagic foci (Fig. 7e, f). In addition, vascular leakage was also detected in Akt1^∆SMC^ mice after injection of FITC-dextran dye (Fig. 7g, h). The effect of Akt1 deficiency on vascular leakage was confirmed by establishing a diabetic retinopathy model by injecting Akt1^∆SMC^ mice with STZ. The blood glucose level increased 4 weeks after STZ injection (Fig. 7i). Vascular permeability was observed in wild-type mice after STZ injection and further increased in Akt1^∆SMC^ mice (Fig. 7j, k). Since Akt1 in VSMCs seems to be important in vascular stability, we assessed the activation of Akt in a type 2 diabetes model, which is vulnerable to vascular stability. Db/Db mice showed high blood glucose levels (Fig. 7l). Staining of the retinas with phospho-Akt antibody showed that Akt activation was significantly suppressed in db/db mice (Fig. 7m, n). Spontaneous hemorrhage was observed in db/db mice (Fig. 7o, p). In addition, the vascular network was severely destroyed and tangled in db/db mice with Akt1^∆SMC^ (Fig. 7q, r). In line with this, vascular permeability was significantly enhanced in db/db mice with Akt1^∆SMC^.

## Discussion

The present study raised three important issues regarding the regulation of angiogenesis: (i) the isoform specificity of Akt, (ii) the paracrine effect of VSMCs, and (iii) the regulatory mechanisms involved in the expression of paracrine factors.

Although Akt isoforms share more than 85% amino acid sequence homology^2^, each isoform has a distinct function in many physiological responses. For example, cell migration is exclusively regulated by Akt1 compared to Akt2^41,42^. On the other hand, insulin-dependent glucose uptake is solely dependent on Akt2 compared to Akt1^35^. The distinctive role of the Akt isoform was demonstrated in gene-targeted mouse models. Mice lacking Akt1 showed a relatively small organism size with a normal blood glucose level, whereas mice lacking Akt2 showed a normal organism size but with type 2 diabetes-like syndrome^3,4^. Similarly, the results also showed that Akt1 predominantly regulated retinal angiogenesis (Fig. 1a–d), tumor angiogenesis (Fig. 2c–f), EC proliferation (Fig. 3a, b), EC sprouting (Fig. 3c, d), Notch3 activation (Fig. 5d, f), and the inhibition of YAP (Fig. 6a–d). The functional specificity of each Akt isoform does not appear to be due to the expression pattern since all three Akt isoforms are expressed in virtually all cells and tissues. Recently, it was reported that the linker region, which is a hypervariable region of the Akt isoform, confers functional specificity in the regulation of cancer cell migration^41^. Moreover, Akt isoforms differentially interact with mammalian target of rapamycin complex 2 (mTORC2)^43^. Finally, phosphoproteomic analysis of EC cells lacking either Akt1 or Akt2 showed that Akt isoforms differentially phosphorylated downstream target substrates^1^. Therefore, Akt1 rather than the Akt2 isoform renders specific functions to regulate retinal angiogenesis.

Although Akt1 in ECs plays a key role in angiogenesis, the role of Akt1 in VSMCs remains unknown. Many studies have shown that the recruitment of pericytes to the endothelium is important for vascular stabilization and proper angiogenesis^44^. Our results raise two issues: (i) ECs are activated only in the presence of Akt1 in VSMCs; and (ii) the recruitment to and coverage by NG2-positive pericytes of the endothelium were downregulated in Akt1^∆SMC^ mice. Several lines of evidence support the critical role of Akt1 in VSMCs in the activation of ECs. For example, strengthening the Akt signaling cascade by deleting one copy of the PTEN allele in VSMCs facilitated EC activation (Fig. 1e–g). Mice lacking Akt1 in VSMCs also showed inhibition of EC proliferation (Fig. 3a, b). Furthermore, EC sprouting was significantly delayed in aortic rings from Akt1^∆SMC^ mice, even in the absence of direct contact with VSMCs (Fig. 3c, d). Therefore, it is reasonable to suggest that Akt1-dependent expression of paracrine factors affects the activation of ECs. Indeed, our results showed that Akt1 regulates the expression of Ang1 and Ang2 in VSMCs (Figs. 5 and 6). It is also noteworthy that the locomotion and differentiation of pericytes into VSMCs are involved in the physiological process of pericyte recruitment and VSMC coverage. Since our results showed that Akt1 regulates the expression of Ang1, Ang2, and possibly other paracrine factors that induce pericyte recruitment, it is possible that Akt1 in preexisting VSMCs may affect the expression of paracrine factors that regulate pericyte recruitment. It is also noteworthy that Akt1 regulates the differentiation of synthetic VSMCs, presumably pericyte cells^34^. Therefore, it is possible that the loss of Akt1 may affect the differentiation of pericytes into VSMCs or the maintenance of mature VSMCs, thereby affecting VSMC coverage. Thus, it is reasonable to suggest that the loss of Akt1 in VSMCs results in the inhibition of VSMC differentiation and expression of paracrine factors, thereby inducing defective coverage and pericyte recruitment to the endothelium.

It has been reported that Ang1 regulates angiogenesis by binding to its cognate receptor Tie2^45^. Ang1 appears to be primarily involved in vascular stabilization since Ang1 transgenic mice showed resistance to vascular leakage^46^. Ang2 shares Tie2 with Ang1 as a receptor; however, the physiological response is different. For instance, Ang2 suppresses Ang1-induced Tie2 activation, and transgenic overexpression of Ang2 disrupts vessel formation in vivo^47^. Therefore, the balance between Ang1 and Ang2 levels seems to be important during angiogenesis. It has been reported that both Ang1 and Ang2 are expressed in ECs and pericytes^19^. In addition, single-cell RNA-sequencing analysis showed that an excess amount of Ang1 and Ang2 was expressed in VSMCs and pericytes (Supplementary Fig. 2). Akt1 might regulate Ang1 and Ang2 expression in VSMCs since the presence of Akt1 is required for Ang1 expression and suppression of Ang2 expression (Fig. 4). In addition, supplementation with an Ang1 neutralizing antibody and Ang2 suppressed angiogenic sprouting in blood vessels isolated from wild-type mice, whereas abrogated angiogenic sprouting of blood vessels from Akt1^∆SMC^ mice was recovered by supplementation with Ang1- or Ang2-neutralizing antibodies (Fig. 7a–d). Therefore, it is reasonable to suggest that Akt1 controls angiogenesis by regulating Ang1 and Ang2 expression in VSMCs. Since it has been reported that mice lacking Akt1 in ECs showed defective angiogenesis^1^, it is also reasonable to assume that the presence of Akt1 in both ECs and VSMCs is required for the regulation of Ang1/2 expression as well as angiogenesis.

Notch3 is expressed in mural cells and is required for the maturation of VSMCs^21^. Loss of Notch3 results in impaired retinal vascularization and the progressive loss of mural cell coverage^48^. A recent report suggested that Notch3 is activated by Akt and regulates the tumor progression of mesenchymal colorectal cancer^49^. In line with this, our results showed that the loss of Akt1 in VSMCs impaired pericyte recruitment as well as VSMC coverage (Fig. 3e–h). In addition, transcriptional activity and activation of Notch3 were significantly affected by Akt1 (Fig. 5a–g). More importantly, Notch3 regulated Ang1 expression (Fig. 5h–o). Therefore, Akt1 might regulate the maturation of blood vessels by Ang1 expression through the activation of Notch3.

Recent evidence supports the idea that the Hippo signaling cascade is involved in regulating angiogenesis^30^. YAP, which is one of the Hippo signaling cascades, is regulated by the PI3K/Akt signaling cascade^50^. Indeed, our results also showed that the nuclear localization and transcriptional activity of YAP were significantly suppressed by Akt1 (Fig. 6a–d). Currently, various transcriptional targets of YAP during angiogenesis have been identified^51^. Since Akt1 regulates the transcriptional activity of YAP as well as the expression of Ang2 (Figs. 4 and 6), it is possible that Ang2 expression might be regulated by YAP. In line with this hypothesis, the expression of Ang2 was significantly regulated by YAP (Fig. 6e–k). An additional report supports that YAP transcriptional activity regulates the expression of Ang2 in ECs^52^. Therefore, it is reasonable to suggest that the expression of Ang1 and Ang2 in VSMCs is mediated by the transcriptional activation of Notch3 by Akt1 and the transcriptional suppression of YAP by Akt1, respectively.

Akt activation was significantly downregulated under diabetic conditions (Fig. 7m, n). It is noteworthy that the level of insulin, which is required for PI3K/Akt to mediate major signaling cascades, is extremely low in type 1 diabetic conditions. As a result, it is possible that the activation of Akt would be low in type 1 diabetic conditions. Dysregulation of insulin signaling in type 2 diabetic conditions seems to be more complicated. Chronic excessive energy conditions are one of the most important preconditions that induce insulin resistance and hyperinsulinemia. Under hyperinsulinemia conditions, downregulation of insulin receptor substrate 2 (IRS2) led to impairment of the PI3K/Akt signaling cascade in metabolic tissues such as muscle and liver^53^. Our results also showed that Akt activation was significantly abrogated in vessels from type 2 diabetic mice (Fig. 7m, n). In addition, loss of Akt1 in VSMCs manifested more severe vascular leakage in the type 2 diabetic background (Fig. 7q, r). Therefore, it is reasonable to suggest that type 1 or type 2 diabetic conditions lead to impaired Akt1 activation, subsequent dysregulation of Notch3 and YAP, imbalanced expression of Ang1 and Ang2 in VSMCs, and unstable blood vessel structure as well as vascular leakage.

In the present study, we suggest the important role of the Akt1 isoform in VSMCs. Akt1 plays a crucial role in pericyte recruitment and coverage as well as in the activation of ECs by the regulation of paracrine factor expression, such as Ang1 and Ang2. Akt1 seems to enhance vascular maturation by upregulating Ang1 through Notch3 activation and downregulating Ang2 through suppression of YAP transcriptional activity. The consequence of a lack of Akt1 activation or Akt1 expression would result in inappropriate vascular maturation that eventually leads to vascular leakage.

## Supplementary information

Supplementary figures
