# Supplementary material for: Akt1-dependent expression of angiopoietin 1 and 2 in vascular smooth muscle cells leads to vascular stabilization
Source: Exp Mol Med. 2022 Aug 5;54(8):1133–45. doi: 10.1038/s12276-022-00819-8 (PMC9440121; doi:10.1038/s12276-022-00819-8)
Supplement: Supplementary file 1 — Supplementary figures [file 12276_2022_819_MOESM1_ESM.pdf]

Supplementary Figure Legends

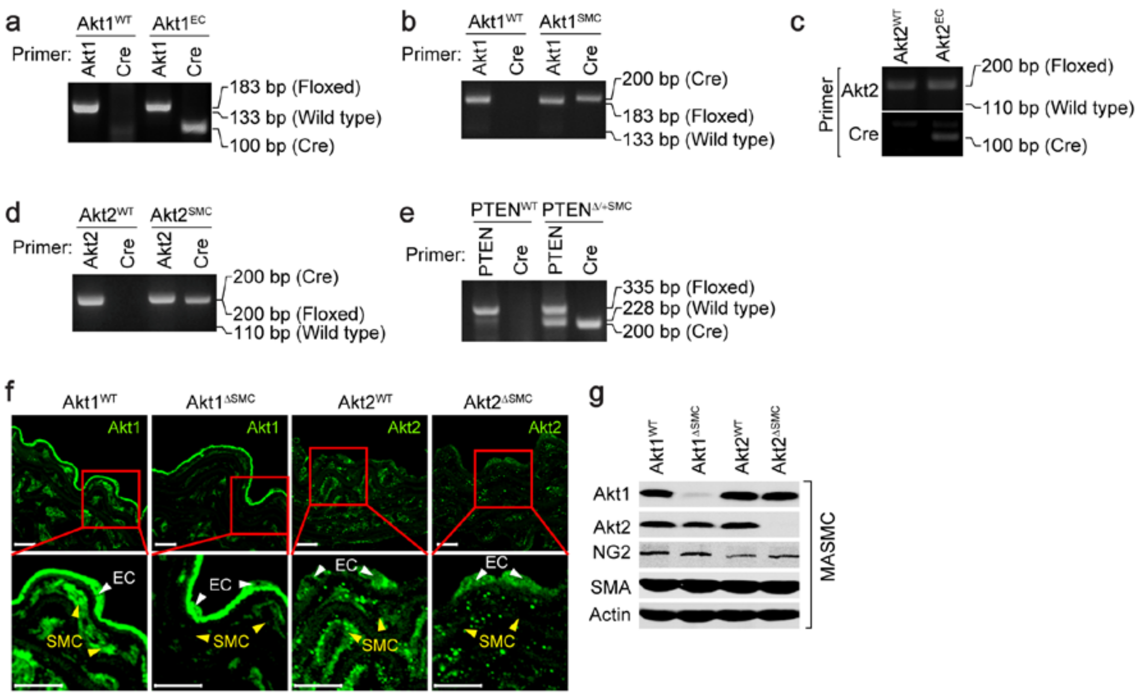

Ha et al. Supplemental Figure S1

Supplementary Fig. 1 Expression of Akt1 and Akt2 in Akt1$^{\Delta SMC}$ and Akt2$^{\Delta SMC}$ mice. a-e Tail tissues of mice were lysed and amplified using specific primers. **f** Aortas were isolated from Akt1^{WT}, Akt1$^{\Delta SMC}$, Akt2^{WT}, and Akt2$^{\Delta SMC}$ mice and stained with Akt1 (green) and Akt2 (green) (n = 4-5). Images were visualized under a confocal microscope. White arrowheads indicated ECs, and yellow arrowheads indicated SMCs. Bar, 10 μm. **g** MASMCs were isolated from Akt1^{WT}, Akt1$^{\Delta SMC}$, Akt2^{WT}, and Akt2$^{\Delta SMC}$ mice and the expression levels of Akt1, Akt2, and VSMC marker genes were assessed by western blot analysis.

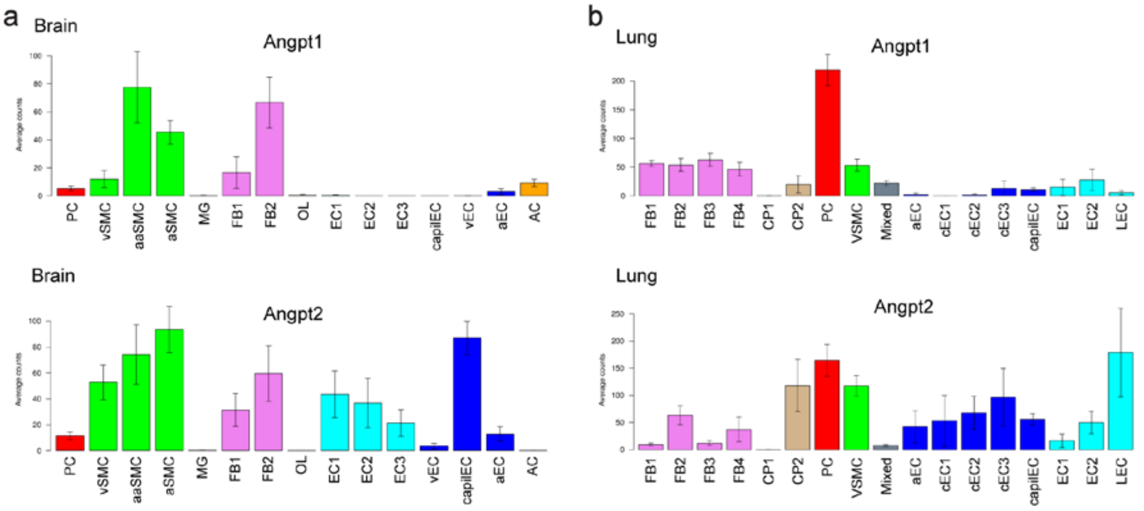

Ha et al. Supplemental Figure S2

Supplementary Fig. 2 Both Ang1 and Ang2 are expressed in VSMCs and pericytes. The expression of both Ang1 and Ang2 was analyzed using single-cell RNA sequencing data base from brain (**a**) and lung (**b**) to ensure the expression of Ang1 and Ang2 in VSMCs and pericytes.

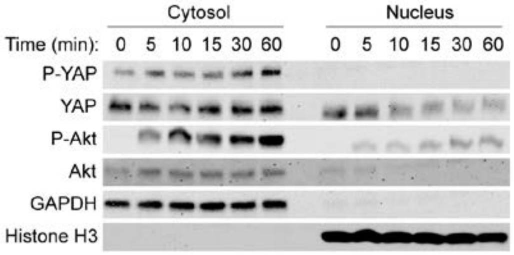

Ha et al. Supplemental Figure S3

Supplementary Fig. 3 Translocation of YAP from nuclear to cytoplasm by PDGF-BB stimulation. VSMCs were stimulated with PDGF-BB in a time-dependent manner. Cells were lysed using nuclear and cytoplasmic extraction reagents and phosphorylation and nuclear localization of YAP and Akt were verified by western blot analysis. GAPDH and Histone H3 were used as markers for cytosol and nucleus, respectively.
